# Quantitative Assessment of Myocardial Perfusion in Physiological and Pathological Hypertrophy Using Myocardial Contrast Echocardiography

**DOI:** 10.1002/clc.70354

**Published:** 2026-05-19

**Authors:** Sadaf Khan, Hammad Saif, Javeria Iqbal

**Affiliations:** ^1^ Khyber Medical College Peshawar Peshawar Pakistan; ^2^ Women Medical and Dental College Abbottabad Abbottabad Pakistan

Dear Editor,

We read with great interest the article by Liu et al. titled “Quantitative Assessment of Myocardial Perfusion in Physiological and Pathological Hypertrophy Using Myocardial Contrast Echocardiography” (Clinical Cardiology, 2026) [1], and we commend the authors for their valuable work on using myocardial contrast echocardiography (MCE) to discriminate between physiological and pathological hypertrophy.

However, we wish to raise a specific methodological concern regarding potential confounding from antihypertensive medications in the pathological hypertrophy group. The authors acknowledge in their Discussion that “some patients in the pathological hypertrophy group received relevant pharmacological treatment, which may have potentially influenced the observed myocardial perfusion parameters.” Yet no systematic data are provided on medication types, dosages, duration, or compliance.

This omission is scientifically consequential because antihypertensive agents have well‐documented and differential effects on myocardial perfusion. Buus et al. demonstrated that angiotensin‐converting enzyme (ACE) inhibition increases coronary reserve and regresses left ventricular hypertrophy, whereas beta‐blockade reduces hyperemic myocardial perfusion [2]. Similarly, Komatsu et al. showed that valsartan, an angiotensin II receptor blocker, increases myocardial blood volume in association with regression of left ventricular hypertrophy in hypertensive patients [3]. Zoghbi et al. further emphasized that antihypertensive medications can substantially modify myocardial perfusion imaging results, complicating the interpretation of observed deficits [4].

Furthermore, the landmark study by Indermühle et al., which the authors cite, provided a validated MCE‐derived cutoff (relative myocardial blood volume < 0.114 mL/mL) for differentiating hypertensive heart disease from athlete's heart using a well‐controlled cohort with documented medication status [5]. Unlike that study, the present investigation proposes a novel diagnostic cutoff of A > 8.13 dB derived from the same cohort used to identify group differences, without internal cross‐validation or external validation [4]. Consequently, the reported sensitivity (92.7%) and specificity (73.5%) are likely overoptimistic.

Nakajima et al. also demonstrated that hypertension per se impairs myocardial blood perfusion reserve independently of left ventricular hypertrophy [6], underscoring the need to account for blood pressure control in the pathological group.

Without detailed medication data, it remains unclear whether the lower A‐values and myocardial blood flow (MBF) observed reflect the pathophysiology of hypertensive hypertrophy itself, the effects of specific drug classes, or incomplete blood pressure control. This potential confounder may bias the ROC analysis and the proposed A > 8.13 dB cutoff.

We respectfully suggest that the authors perform a sensitivity analysis limited to untreated hypertensive patients (if available) or adjust for medication class and blood pressure control using ANCOVA. Independent validation of the A > 8.13 dB cutoff in a separate prospective cohort is also necessary before it can be recommended for clinical use. Reporting these data would substantially strengthen the conclusion that MCE‐derived A‐values reliably discriminate physiological from pathological hypertrophy independent of treatment effects.

## Author Contributions

The authors have read and approved the final version of this letter.

## Funding

The authors have nothing to report.

## Ethics Statement

The authors have nothing to report.

## Consent

The authors have nothing to report.

## Conflicts of Interest

The authors declare no conflicts of interest.

## Data Availability

The authors have nothing to report.
